# Assessment of quality of life in Gaucher disease: A methodological approach

**DOI:** 10.1002/mgg3.1549

**Published:** 2020-11-21

**Authors:** Vitória S. Zizemer, Tatiele Nalin, Ida Vanessa D. Schwartz, Ana Paula Vanz

**Affiliations:** ^1^ Universidade Federal do Rio Grande do Sul Porto Alegre Brazil; ^2^ Postgraduate Program in Medicine: Medical Sciences Universidade Federal do Rio Grande do Sul Porto Alegre Brazil; ^3^ Department of Genetics Universidade Federal do Rio Grande do Sul Porto Alegre Brazil; ^4^ Medical Genetics Service Hospital de Clínicas de Porto Alegre Porto Alegre Brazil; ^5^ Nurse Department Faculdades Integradas de Taquara Taquara Brazil

## Abstract

This letter discusses the use of Lansky Score as an instrument for assessing quality of life in Gaucher Disease.

Dear Editor,

We read with great interest the article by Cerón‐Rodríguez et al. (2017), titled “Improvement of life quality measured by Lansky Score after enzymatic replacement therapy in children with Gaucher disease type 1,” which reported improvement in quality of life (QoL) among children with Gaucher disease type 1 (GD1) as measured by the Lansky score and concluded that these patients achieved improvement in QoL after 6, 12, 18, 24, and 30 months of enzyme replacement therapy (ERT).

There is no doubt that the ERT did cause substantial improvement in the systemic manifestations of GD in those patients. However, we have some concerns about the paper. The first one refers to the assumption that patients presented type 1 of the disease. It would be important that authors clarify which parameters they used for that classification, especially considering that patients two to four were homozygous for the p.Leu483Pro variant, a genotype which is well known to be associated with Gaucher types 2 or 3.

Another concern refers to the instrument used by the authors for QoL evaluation. According to the original article that first proposed the Lansky score, the evaluated children–all patients with cancer undergoing chemotherapy––and control participants had the same functional capacity (Lansky et al., 1987). The authors further suggested that children with cancer were able to resume most of their usual activities after completion of treatment, and that parents reported that the convalescence period had been brief. Considering also that, even in the 1970s, a few years before the Lansky score was first proposed, the combined 5‐year survival rate for childhood cancers was already 58% (rising to 83% between 2005 and 2011) (American Cancer Society, 2016) and that in many cases, cancer in children presents as a transient, curable disease, any comparison between these patients and children with GD1––a chronic, incurable disease that requires lifelong treatment and may, thus, have a greater impact on QoL––is questionable at best.

Furthermore, the Lansky score primarily evaluates functional performance, using data such as the activity level of play as observed by parents (Lansky et al., 1987); however, the article in question included a 12‐month‐old child, an age during which the development process is still in full swing. This calls into question whether parents were truly able to distinguish improvements resulting from the treatment process from normal development milestones. Apparently, the Lansky scale overlaps, at least partly, with the natural child developmental process, which may be another source of bias.

We would like to raise some questions to Cerón‐Rodriguez et al. to better interpret the results of the work. Regarding the patient who was 4 years old and especially the 10‐year‐old, are they attending school or pre‐school successfully? How were the infants in relation to the pre‐ERT child developmental stages? Did the parents have other children to whom the patients could have been compared to? Were they experienced parents or novices?

Cerón‐Rodríguez et al. clearly state that there is no ideal questionnaire for assessment of QoL in patients with Gaucher disease, as a specific instrument is not available. Nevertheless, we wonder whether the data provided by the instrument chosen by the authors can be used to support their claim of an improvement in QoL, or whether it can only demonstrate an improvement in the functional ability of these patients. Finally, we wonder whether the authors would recommend use of the Lansky score in future studies of this population.

## CONFLICT OF INTEREST

The authors declare that there is no conflict of interest.

## AUTHOR'S CONTRIBUTION

This letter was written in collaboration by the authors.

## References

[mgg31549-bib-0001] American Cancer Society . (2016). Cancer Facts & Figures 2016. American Cancer Society. https://www.cancer.org/research/cancer‐facts‐statistics/all‐cancer‐facts‐figures/cancer‐facts‐figures‐2016.html

[mgg31549-bib-0002] Cerón‐Rodríguez, M. , Barajas‐Colón, E. , Ramírez‐Devars, L. , Gutiérrez‐Camacho, C. , & Salgado‐Loza, J. L. (2017). Improvement of life quality measured by Lansky Score after enzymatic replacement therapy in children with Gaucher disease type 1. Molecular Genetics & Genomic Medicine, 6(1), 27–34. 10.1002/mgg3.339 29471591 PMC5823673

[mgg31549-bib-0003] Lansky, S. B. , List, M. A. , Lansky, L. L. , Ritter‐Sterr, C. , & Miller, D. R. (1987). The measurement of performance in childhood cancer patients. Cancer, 6, 1651–1656. 10.1002/1097-0142(19871001)60:7<1651:aid-cncr2820600738>3.0.co;2-j 3621134

